# Estimation of bovine leukemia virus (BLV) proviral load harbored by lymphocyte subpopulations in BLV-infected cattle at the subclinical stage of enzootic bovine leucosis using BLV-CoCoMo-qPCR

**DOI:** 10.1186/1746-6148-9-95

**Published:** 2013-05-04

**Authors:** Carlos Javier Panei, Shin-nosuke Takeshima, Takashi Omori, Tetsuo Nunoya, William C Davis, Hiroshi Ishizaki, Kazuhiro Matoba, Yoko Aida

**Affiliations:** 1 Viral Infectious Diseases Unit, RIKEN, 2-1 Hirosawa, Wako, Saitama, 351-0198, Japan; 2 CONICET and Virology Laboratory, Faculty of Veterinary Sciences, National University of La Plata, La Plata, Argentina; 3 Nippon Institute for Biological Science, 9-2221-1 Shinmachi Ome, Tokyo, 198-0024, Japan; 4 Department of Veterinary Microbiology and Pathology, Washington State University, Pullman, WA, 99164-7040, USA; 5 NARO Institute of Livestock and Grassland Sciences, 768 Senbonmatsu, Nasushiobara, Tochigi, 329-2793, Japan

**Keywords:** Bovine leukemia virus (BLV), Proviral load, BLV-CoCoMo-qPCR, CD5^+^IgM^+^ B cell, Cell sorting, Flow cytometry

## Abstract

**Background:**

Bovine leukemia virus (BLV) is associated with enzootic bovine leukosis (EBL), which is the most common neoplastic disease of cattle. BLV infection may remain clinically silent at the aleukemic (AL) stage, cause persistent lymphocytosis (PL), or, more rarely, B cell lymphoma. BLV has been identified in B cells, CD2^+^ T cells, CD3^+^ T cells, CD4^+^ T cells, CD8^+^ T cells, γ/δ T cells, monocytes, and granulocytes in infected cattle that do not have tumors, although the most consistently infected cell is the CD5^+^ B cell. The mechanism by which BLV causes uncontrolled CD5^+^ B cell proliferation is unknown. Recently, we developed a new quantitative real-time polymerase chain reaction (PCR) method, BLV-CoCoMo-qPCR, which enabled us to demonstrate that the proviral load correlates not only with BLV infection, as assessed by syncytium formation, but also with BLV disease progression. The present study reports the distribution of BLV provirus in peripheral blood mononuclear cell subpopulations isolated from BLV-infected cows at the subclinical stage of EBL as examined by cell sorting and BLV-CoCoMo-qPCR.

**Results:**

Phenotypic characterization of five BLV-infected but clinically normal cattle with a proviral load of > 100 copies per 1 × 10^5^ cells identified a high percentage of CD5^+^ IgM^+^ cells (but not CD5^-^ IgM^+^ B cells, CD4^+^ T cells, or CD8^+^T cells). These lymphocyte subpopulations were purified from three out of five cattle by cell sorting or using magnetic beads, and the BLV proviral load was estimated using BLV-CoCoMo-qPCR. The CD5^+^ IgM^+^ B cell population in all animals harbored a higher BLV proviral load than the other cell populations. The copy number of proviruses infecting CD5^-^ IgM^+^ B cells, CD4^+^ cells, and CD8^+^ T cells (per 1 ml of blood) was 1/34 to 1/4, 1/22 to 1/3, and 1/31 to 1/3, respectively, compared with that in CD5^+^ IgM^+^ B cells. Moreover, the BLV provirus remained integrated into the genomic DNA of CD5^+^ IgM^+^ B cells, CD5^-^ IgM^+^ B cells, CD4^+^ T cells, and CD8^+^ T cells, even in BLV-infected cattle with a proviral load of <100 copies per 10^5^ cells.

**Conclusions:**

The results of the recent study showed that, although CD5^+^ IgM^+^ B cells were the main cell type targeted in BLV-infected but clinically normal cattle, CD5^-^ IgM^+^ B cells, CD4^+^ cells, and CD8^+^ T cells were infected to a greater extent than previously thought.

## Background

Bovine leukemia virus (BLV), a close relative of human T cell leukemia virus types-1 and -2 (HTLV-1 and HTLV-2), is the etiologic agent responsible for enzootic bovine leukosis (EBL), which is the most common neoplastic disease of cattle. Infection by BLV may remain clinically silent at the aleukemic (AL) stage. However, in 30% of infected cattle the infection may manifest as persistent lymphocytosis (PL; a condition characterized by an increase in the number of B lymphocytes), and in around 1–5% of cases it may manifest as B cell lymphoma after a long period of latency [1]. Sheep experimentally inoculated with BLV develop B cell tumors at a higher frequency than naturally infected cattle, and the period of latency is shorter [2,3].

In infected cattle with no evident tumor, BLV has been identified in B cells, CD2^+^ T cells, CD3^+^ T cells, CD4^+^ T cells, CD8^+^ T cells, γ/δ T cells, monocytes and granulocytes [4-6]. By contrast, the increase in lymphocyte numbers observed in cows with PL is entirely attributable to the expansion of the CD5^-^ and CD5^+^ B cell subpopulations, indicating that CD5^-^ and CD5^+^ B cells are the only mononuclear cells within the peripheral blood that are significantly infected with BLV [5]. Furthermore, the most consistent tumor cell phenotypes isolated from cattle with EBL are CD5^+^, CD6^–^, B1 low^+^, B2^+^, major histocompatibility complex class II^+^, and either sIgM^+^ or sIgM^–^; this indicates the involvement of the CD5^+^ B cell sub population rather than the CD5^-^B cell sub population. However, the mechanism by which BLV induces uncontrolled CD5^+^ B cell proliferation is unknown. It is interesting to note that in sheep, transformed B cells show a CD5^-^ phenotype [7]. Indeed, we previously showed that the extended survival of peripheral blood mononuclear cells (PBMCs) *ex vivo* was mainly due to the presence of BLV-expressing CD5^–^ B cells, indicating that sheep CD5^–^ B cells may be particularly susceptibility to the transforming effects of BLV [8]. This increase in the survival of BLV-expressing sheep PBMCs was also associated with an increase in the expression of mRNA for *bcl-xl,* but not that for *bcl-2* or *bax*[9]. However, the mechanism by which BLV protects *ex vivo* cultured cells against apoptosis is unknown.

After infecting cattle, BLV enters a period of latency, during which expression is blocked at the transcriptional level [10-12]. BLV-infected cattle retain at least one copy of the full-length proviral genome throughout the course of the disease [13], suggesting that the BLV provirus remains integrated within the cellular genome [10], even in the absence of detectable BLV antibodies [14]. Therefore, diagnostic BLV polymerase chain reaction (PCR) techniques, which detect the integrated BLV proviral genome within the host genome, are now commonly used to detect BLV infection in addition to routine diagnostic tests such as agar gel immunodiffusion and enzyme-linked immunosorbent assays (ELISAs) [13,15-18]. Recently, we developed a new quantitative real-time PCR method using Coordination of Common Motifs (CoCoMo) primers to measure the proviral load of both known and novel BLV variants in BLV-infected animals [14,19]. The assay was highly effective in detecting BLV in cattle from a number of international locations. The BLV-CoCoMo-qPCR technique amplifies a single-copy host gene, the *bovine leukocyte antigen (BoLA)-DRA* gene, in parallel with viral genomic DNA, which effectively normalizes the level of viral genomic DNA. Thus, we were able to show that the proviral load correlates not only with the level of BLV propagation, as assessed by syncytium formation, but also with BLV disease progression.

While the primary cellular target of BLV is B cells, recent studies suggest that monocytes, granulocytes, CD2^+^ T cells, CD3^+^ T cells, CD4^+^ T cells, CD8^+^ T cells and γ/δ T cells are also targets [4-6,20]. However, because Mirsky et al. [5], fractionated B cells into the CD5^+^ IgM^+^ B cells and CD5^-^ IgM^+^ B cell subpopulations, but did not fractionate CD2^+^ T cells into the CD4^+^ and CD8^+^ T cell subpopulations. In contrast, Wu et al. [21] isolated the CD4+ and CD8+ T cell subpopulations, but did not fractionate B cells into the CD5^+^ IgM^+^ B cells and CD5^-^ IgM^+^ B cell subpopulations. It remains to be clarified the variations of the BLV proviral load among CD5^+^ IgM^+^ B cells, CD5^-^ IgM^+^ B cells, CD4^+^ T cells, and CD8^+^ T cells in the same experiment. Therefore, to clarify whether these subpopulations are susceptible to BLV infection, we obtained PBMCs from cattle naturally infected with BLV and isolated CD5^+^ IgM^+^ B cells, CD5^-^ IgM^+^ B cells, CD4^+^ T cells, and CD8^+^ T cells by flow cytometry or using magnetic beads. We then estimated the BLV proviral load using the BLV-CoCoMo-qPCR technique. The results show that CD5^+^ IgM^+^ B cells, CD5^-^ IgM^+^ B cells, CD4^+^ T cells, and CD8^+^ T cells are all primary targets for BLV.

## Methods

### Animals, blood samples, sera, and DNA extraction

Blood samples were obtained from eight Holstein cows (N790, N791, N818, N789, N787, N733, N823, and N788) and one Japanese black cow (O10) in Japan (Table 1). PBMCs were separated according to the method of Miyasaka and Trunka [22]. Serum was also obtained from the same cows. The subclinical stage of BLV infection was evaluated according to the lymphocyte count (cells per μl) and the age of each cow (≤8,500 = normal and ≥13,000 = lymphocytosis for cows aged 2–3 years; ≤5,500 = normal and ≥7,500 = lymphocytosis for cows aged ≥6 years), and by detecting atypical mononuclear cells [23]. In the PL case, three separate lymphocyte counts were performed at different times. All experiments were conducted in accordance with the Guidelines for Laboratory Animal Welfare and Animal Experiment Control set out by the Nippon Institute for Biological Science (Permit number: 12Kenkyu-50).

**Table 1:** Outline of examined BLV-infected but clinically normal cattle

**Cattle**	**Age (years)**	**WBC (/μl)**	**Lymphocyte (/μl) (%)**	**Clinical stage**^**a**^	**ELISA**^**b**^	**Proviral load**^**c**^ **(Copies/1 × 10**^**5**^**cells)**	**Syncytium assay**^**d**^ **(Number/5 × 10**^**6**^**cells)**
BLV-free normal cattle							
N790	2.5	9,540	6,382 (66.9)	-	-	0	0
N791	2.5	8,260	4,956 (60.0)	-	-	0	0
BLV-infected cattle							
1) Cattle with < 100 proviral load							
N818	2.0	9,410	5,147 (54.7)	AL	+	8	0
N789	2.5	5,472	5,472 (60.2)	AL	+	26	1
2) Cattle with > 100 proviral load							
N787 N733	2.5	8,730	4,487 (51.4)	AL	+	294	250
	2.5	8,230	6,313 (68.4)	AL	+	1,614	400
N823	2.0	9,340	6,239 (66.8)	AL	+	11,112	11,000
N788	2.5	9,620	5,435 (56.5)	AL	+	18,094	17,500
O10	15.0	12,200	7,900 (64.8)	PL	+	10,689	9,086

^a^The clinical stage of BLV infection was evaluated according to the lymphocyte count (per 1 μl), the detection of atypical mononuclear cells, and the age of the animal [19]. AL, BLV-infected but clinically and hematologically normal cattle; PL, BLV-infected but clinically normal cattle showing an increase in the number of apparently normal B lymphocytes. In the latter case, three separate lymphocyte counts were performed at different times and all yielded the same results.

^b^ELISA was performed using an anti-BLV ELISA kit according to the manufacturer’s instructions (JNC Inc., Tokyo, Japan). +, positive for anti-BLV antibodies; -, negative for anti-BLV antibodies.

^c^The proviral load (expressed as the copy number per 10^5^ peripheral blood mononuclear cells [PBMCs]) was evaluated by BLV-CoCoMo-qPCR as previously described [19].

^d^PBMCs were mixed with CC81 cells and 4 μg/ml polybrene (Sigma, St. Louis, MO) and used in the syncytium formation assay [19]. Syncytia were counted under a light microscope.

Genomic DNA was isolated from EDTA-treated whole blood samples using the Wizard Genomic DNA Purification Kit (Promega Corporation, Tokyo, Japan) and subsequently used for PCR.

### Measurement of the BLV proviral load using BLV-CoCoMo-qPCR

The BLV proviral load was measured using BLV-CoCoMo-qPCR as previously described [19]. Briefly, the BLV long terminal repeat (LTR) region was amplified using the degenerate primer pair: CoCoMo 6 and CoCoMo 81. FAM BLV was used as a probe. The *BoLA-DRA* gene (internal control) was amplified using the primer pair, DRA643 and DRA734. VIC-DRA was used as a probe.

### ELISA

An anti-BLV antibody ELISA kit (JNC Inc., Tokyo, Japan) was used to detect anti-BLV antibodies according to the manufacturer’s instructions.

### Syncytium formation assay

To determine the presence of BLV in the cattle, PBMCs (5 × 10^6^ cells/4 ml) were mixed with CC81 cells (cat cells transformed with mouse sarcoma virus;1 × 10^5^ cells/4 ml) and 4 μg/ml polybrene (Sigma, St. Louis, MO) in 6 cm diameter culture dishes and used in a syncytium formation assay [19,24]. Syncytia were counted under a light microscope after staining with May-Grunwald Giemsa. CC81 cells were maintained in RPMI-1640 medium supplemented with 10% heat-inactivated fetal bovine serum (FBS), penicillin (100 U/ml), and streptomycin (100 μg/ml).

### Monoclonal antibodies (MAbs) and detection of surface markers by flow cytometry

PBMCs were labeled using optimal concentrations of the following MAbs: CACT105A (mouse anti-bovine CD5; VMRD Inc., Pullman, WA); BAQ44A (mouse anti-bovine IgM; VMRD Inc.); ILA11A (mouse anti-bovine CD4; VMRD Inc.); or 7C2B (mouse anti-bovine CD8; VMRD Inc.). The cells were then stained with the following secondary fluorophore-labeled MAbs: allophycocyanin (APC)-conjugated rat anti-mouse IgG1 (BD Pharmingen, Tokyo, Japan) to detect CACT105A-positive cells; phycoerythrin (PE)-conjugated goat anti-mouse IgM (CALTAG Laboratories, Carlsbad, CA) to detect BAQ44A-positive cells; or PE-conjugated goat anti-mouse IgG2a (Invitrogen, Camarillo, BD Japan) to detect ILA11A- or 7C2B-positive cells. After staining, cells were analyzed using a FACSCalibur™ flow cytometer (BD Japan, Tokyo, Japan) and the data were analyzed using FCS Express (Ver. 3; De Novo Software, Los Angeles, CA). Cells stained with normal mouse serum and appropriate secondary antibodies were used as a negative control.

### Cell sorting

CD5^+^ IgM^+^ cells and CD5^+^ IgM^-^ cells were separated from PBMCs using a BD FACSAria™ cell sorter (BD Japan). The purity of the sorted populations was assessed using the same cytometer.

CD4^+^ and CD8^+^ cells were purified using the MACS® System (Miltenyi Biotech Inc, Auburn, CA). In brief, PBMCs were stained with ILA11A MAb (mouse anti-bovine CD4; VMRD Inc.) or 7C2B MAb(mouse anti-bovine CD8; VMRD Inc.) and captured by an anti-mouse IgG MAb conjugated to magnetic beads. Magnetic bead-bound cells were separated on an LS column. The purity of the CD4^+^ and CD8^+^ cell populations was calculated by indirect immunofluorescence analysis.

## Results

To estimate the BLV proviral load in cattle at the clinically normal stage of BLV infection, we obtained blood samples from eight Holstein cows and one Japanese black cow and analyzed them by BLV-CoCoMo-qPCR. Two cattle were BLV-negative (N790 and N791) and seven were BLV-positive (N818, N789, N787, N733, N823, N788 and O10) (Table 1). The same results were obtained using the anti-BLV ELISA. The seven BLV-infected cattle were then classified according to the EC-leukosis key [23]: Six cattle were categorized as BLV-infected but clinically and hematologically normal cattle, and one was categorized as BLV-infected but clinically normal cattle with PL.

The BLV proviral load in the nucleated cells isolated from blood samples taken from the nine cattle was examined using BLV-CoCoMo-qPCR, and the infection capacity of BLV was assessed using the syncytium assay (Table 1). The proviral load correlated strongly with the syncytium count. For example, the proviral load in animals N787, N733, N823, N788 and O10 ranged from 294 to 18,094 copies per 10^5^ cells, and syncytium numbers ranged from 250 to 17,500 per 5 × 10^6^ PBMCs. Furthermore, animals N818 and N789, which had low proviral loads (8 and 26 copies per 10^5^ PBMCs, respectively) also had very low syncytium counts (0 and 1 per 5 × 10^6^ PBMCs, respectively), meaning that the sensitivity of CoCoMo-qPCR for detecting BLV infection was greater than that of the syncytium assay.

**Figure 1:**
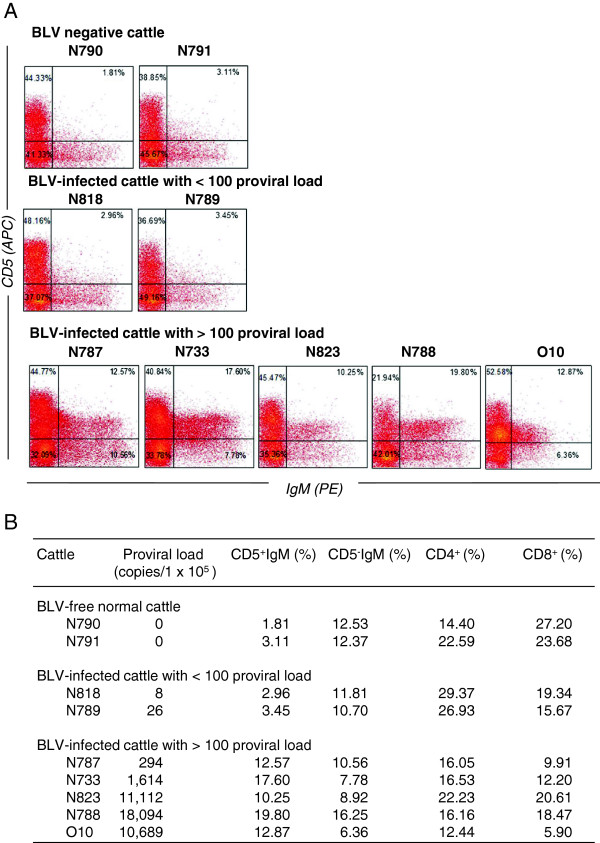
**Flow cytometric analysis of PBMCs isolated from BLV-negative and BLV-positive cattle with proviral loads of <100 and > 100 copies per 10**^**5 **^**cells.** (**A**) Dual color flow cytometric analysis of PBMCs isolated from BLV-negative cattle (animals N790 and N791) and BLV-infected cattle with proviral loads < 100 copies per 10^5^ cells (animals N818, N789) and > 100 copies per 10^5^ cells (animals N787, N733, N823, N788 and N10). Each profile was separated into four quadrants on the basis of control staining (cells were incubated with normal mouse serum and then stained with appropriate secondary antibodies), which signify single-positive [orange (574 nm) and red (660 nm)], double-negative, or double-positive staining. The values in each gate indicate the percentage of the total PBMC population. (**B**) The proviral load and the percentage of CD5^+^ IgM^+^ cells, CD5^-^ IgM^+^ B cells, CD4^+^ T cells and CD8^+^ T cells in the indicated cattle.

The percentage of CD5^+^ IgM^+^ B cells, CD5^-^ IgM^+^ B cells, CD4^+^ T cells, and CD8^+^ T cells within the PBMC population was determined by flow cytometry (Figure 1). Cattle with a proviral load >100 copies per 10^5^ cells showed an increased percentage of CD5^+^ IgM^+^ B cells (> 10%) compared with BLV-negative cattle or cattle with a proviral load < 100 copies per 10^5^ cells (< 5%). In particular, the percentage of CD5^+^ IgM^+^ B cells was higher than the percentage of CD5^-^ IgM^+^ B cells in cattle with a proviral load >100 copies per 10^5^ cells. By contrast, although the proportion of CD4^+^ T cells in cattle with a proviral load >100 copies per 10^5^ cells except animal O10 was similar to those in BLV-negative cattle, the proportion of CD8^+^ T cells in all BLV-infected cattle was lower than those in BLV-negative cattle.

Next, to assess the tropism of BLV for each lymphocyte subpopulation, animals N733, N788 and O10 (all with a proviral load >100 copies per 10^5^ cells) were selected from the BLV-positive group. Animal N818 was selected to represent cattle with a proviral load <100 copies per 10^5^ cells because < 100 proviral load may be the margin of sensitivity of CoCoMo-qPCR. CD5^+^ IgM^+^ B cells and CD5^-^ IgM^+^ B cells were isolated by flow cytometry and CD4^+^ T and CD8^+^ T cells were purified using the MACS® System (Table 2). All cell populations were > 90.0% pure, except for the CD8^+^ T cells isolated from the blood of animal O10, which were only 75.0% pure because of the low number of CD8^+^ T cells present in this animal. Therefore, there is possibility that results from BLV detection in CD8^+^ T cells from animal O10 could be due to contamination by other cell type. In all the selected cattle, the BLV copy number was higher in CD5^+^ IgM^+^ B cells than in the other lymphocyte populations. As shown in Table 2, CD5^+^ IgM^+^ B cells isolated from animal N733 showed a proviral load approximately 30-fold higher than that in the other lymphocyte populations. The difference was around 3-fold in animal N788, and around 5-fold in animal O10. Despite the fact that animal N818 harbored a low proviral load of which lower than the limitation of sensitivity of CoCoMo-qPCR, we were still able to detect the BLV provirus in all four lymphocyte populations. The copy number in CD5^+^ IgM^+^ B cells was around16-fold higher than in CD4^+^ T cells and CD8^+^ T cells, but was similar (1.7-fold difference) to that in CD5^-^ IgM^+^ B cells.

**Table 2:** Proviral load in the different lymphocyte subpopulations isolated from selected BLV-infected but clinically normal cattle.

**Cattle**	**Cell population**^**a**^	**Purity**^**b**^**(%)**	**Proviral load**^**c**^**(Copies/10**^**5**^**cells)**
1) BLV-infected cattle (proviral load > 100 copies/10^5^ cells)			
N733	CD5^+^ IgM^+^	95.5	40,338
	CD5^-^ IgM^+^	94.1	1,590
	CD4^+^	93.0	1,148
	CD8^+^	95.0	1,144
N788	CD5^+^ IgM^+^	97.4	33,529
	CD5^-^ IgM^+^	98.4	11,258
	CD4^+^	90.0	15,232
	CD8^+^	91.0	11,116
O10	CD5^+^ IgM^+^	97.4	55,449
	CD5^-^ IgM^+^	98.4	9,203
	CD4^+^	90.0	11,254
	CD8^+^	75.0	21,915
2) BLV-infected cattle (proviral load < 100 copies/10^5^ cells)			
N818	CD5^+^ IgM^+^	93.3	97
	CD5^-^ IgM^+^	97.6	54
	CD4^+^	92.3	6
	CD8^+^	97.1	6

^a^CD5^+/-^IgM^+^ cells were sorted using a flow cytometer and the MACS System was used to sort CD4^+^ and CD8^+^ cells.

^b^The purity of the CD5^+/-^IgM + cells was calculated using a flow cytometer based at the Brain Sciences Institute. The purity of the CD4^+^ and CD8^+^ cells obtained using the MACS Separation System was assessed by immunofluorescence analysis.

^c^The proviral load (expressed as the copy number per 10^5^ cells) was estimated using BLV-CoCoMo-qPCR [19].

Finally, we predicted the actual number of proviral copies per ml of peripheral blood in each of the lymphocyte subpopulations using the following equation (Table 3):

$$\begin{array}{l} \text{number}\;\text{of}\;\text{proviral}\;\text{copies}\;\text{in}\;\text{each}\;\text{lymphocyte}\;\text{subpopulation}\\ \text{per}\;\text{ml}\;\text{of}\;\text{blood}=\left(\frac{\text{lymphocytes}}{\mathrm{\mu l}}\right)\times \left(\text{subpopulation}\;\text{rate}\right)\\ \;\times \frac{\text{proviral}\;\text{load}\;\text{in}\;\text{each}\;\text{subpopulation}}{10^{5}}\times 10^{3} \end{array}$$

**Table 3:** **Estimation of the proviral copy number (per 1 mL peripheral blood**^**a**^**) in each lymphocyte subpopulation using BLV-CoCoMo-qPCR**

**Cattle**	**Proviral load (Copies/10**^**5**^**cells)**	**Proviral copy number/ml of blood in each of the lymphocyte subpopulations**			
		**CD5**^**+**^**IgM**^**+**^	**CD5**^**-**^**IgM**^**+**^	**CD4**^**+**^	**CD8**^**+**^
1) BLV-infected cattle ( proviral load > 100 copies/10^5^ cells)					
N733	1,614	268,914	7,809	11,980	8,811
N788	18,094	360,816	99,429	133,782	111,587
O10	10,689	450,514	36,951	88,382	81,626
2) BLV-infected cattle (proviral load < 100 copies/10^5^ cells)					
N818	8	148	328	91	60

^a^The proviral load in each lymphocyte subpopulation (expressed as the copy number per 10^5^ PBMCs) was estimated by BLV-CoCoMo-qPCR [19] and then calculated in terms of copies per ml.

BLV proviruses were mainly present in CD5^+^ IgM^+^ B cells isolated from the three BLV-infected cattle with a proviral load >100 copies per 10^5^ cells (N733, N788 and O10), followed by CD4^+^ T cells; however, the proviral loads in CD4^+^ cells were around 3- to 22-times lower than those in CD5^+^ IgM^+^ B cells. BLV also infected CD8^+^ T cells and CD5^-^ IgM ^+^ B cells, although the proviral loads in each of these subpopulations were different in each animal. In addition, in the case of animal with low proviral load N818 (proviral load <100 copies per 10^5^ cells), the actual proviral copy number in CD5^-^ IgM^+^ B cells per ml blood was greater than that in CD5^+^ IgM^+^ B cells, CD4^+^ T cells, or CD8^+^ T cells.

## Discussion

The present study showed that the proviral load in CD5^+^ IgM^+^ B cells (which are the primary target cells for BLV infection) was higher than that in CD4^+^ T cells, CD8^+^ T cells, or CD5^-^ IgM^+^ B cells, both in terms of proviral load per 10^5^ cells (Table 2) and in terms of actual copy number per ml of blood (Table 3) in all cattle with a proviral load >100 copies per 10^5^ cells. Interestingly, we showed that the second most commonly infected cell in these animals was the CD4^+^ T cell. Furthermore, we showed that BLV appears to infect both CD8^+^ T cells and CD5^-^ IgM ^+^ B cells to a greater extent than was reported in a previous study [5]. The current data support the studies by Williams et al. [20], Stott et al. [4], and Wu et al. [21] which reported that some T cells can be infected by BLV. However, in contrast to our results showing that CD4^+^ T cells are the second most common target for BLV infection in cattle, Schwartz et al. [6] reported that, although B cells, CD8^+^ T cells, monocytes, and granulocytes were infected by BLV, CD4^+^ T cells were not. Moreover, Mirsky et al, used flow cytometry and single-cell PCR to show that CD5^+^ IgM^+^ and CD5^-^ IgM^+^ B cells were the only PBMCs infected with BLV in seropositive cows, either with or without PL [5]. They also suggested that peripheral CD2^+^ T cells, γ/δ T cells, and monocytes are not a major reservoir for BLV infection. By contrast, the present study showed that the BLV provirus remains integrated in the DNA of CD5^+^ IgM^+^ B cells, CD5^-^ IgM^+^ B cells, CD4^+^ T cells, and CD8^+^ T cells, even in animals with a proviral load <100 copies per 10^5^ cells of which lower than the limitation of sensitivity of CoCoMo-qPCR. Thus, we were able to use cell sorting and BLV-CoCoMo-qPCR techniques to detect the BLV provirus in all the lymphocyte subpopulations isolated from BLV-infected, clinically normal, cattle with and without PL. Taken together, the results show that CD5^+^ IgM^+^ B cells, CD5^-^ IgM^+^ B cells, CD4^+^ T cells, and CD8^+^ T cells are all primary targets for BLV.

Thus, it appears that BLV can infect a broad spectrum of cells, although its receptor(s) remains unknown. A previous report used the BLV envelope (ENV) receptor binding domain showed that BLV receptor molecules are expressed by pro/pre B cells (but not by mature/arrested B cells), by activated B and T cells (but not by arrested B and T cells), by human thymus cells induced by IL-7, and by proliferating lymphocytes [25]. CD4, CD8, and CD5^-^ IgM^+^ B cells proliferate in an antigen-specific manner; however, CD5^+^ IgM^+^ B cells can proliferate in the absence of antigen. Therefore, CD5^+^ IgM^+^ B cells may constitutively express receptors for BLV, which may explain why CD5^+^ IgM^+^ B cells harbor a high viral load.

One advantage of measuring proviral loads is that such measurements can be used to follow the dynamics of BLV-infected cells *in vivo*. Mirsky and coworkers [5] reported that the infection rate was approximately 40-fold higher for CD5^+^ B cells than for CD2^+^ T cells (121 ± 244 for CD5^+^ B cells and 3 ± 6 for CD2^+^ T cells), suggesting that B cells are the only PBMCs significantly infected by BLV. However, they did not subfractionate CD2^+^ T cells to CD4^+^ and CD8^+^ cells. Our result showed that CD4^+^ T cells harbored higher proviral copy numbers than CD8^+^ and CD5^-^IgM^+^ B cells in all three cattle with a proviral load >100 copies per 10^5^ cells. In addition, the data showed that the fold difference in the proviral load in CD5^+^ IgM^+^ B cells and T cells (CD4^+^ T cells + CD8^+^ T cells) ranged from 1.5 (360,816/(133,782 + 111,587) copies per ml) in N788 to 13 (268,914/(11,980 + 8,811) copies per ml) in N733. This indicates that, in addition to CD5^+^ IgM^+^ B cells, T cells are also infected with BLV. Moreover, the syncytium assay were used for comparing with PBMC and purified CD4^+^ T cell and rate of the number of syncytium formation with 5 × 10^6^ of PBMC per with CD4^+^ T cells were almost 1.6 (data not shown). This result suggests that CD4^+^ T cells could be infected by BLV, and that BLV-infected CD4^+^T cells may be the source of infectious BLV-infected cells that can then infect other cells. Both the previous studies and the present study examined the proviral load in cell populations derived from peripheral blood. It is still not known which peripheral blood or organs maintain BLV proliferation. To investigate the mechanism(s) underlying BLV proliferation *in vivo*, it will be necessary to analyze the proviral load in key organs and in peripheral blood.

## Conclusions

To clarify which subpopulations of lymphocytes were infected by BLV at subclinical stage, we used the BLV-CoCoMo-qPCR method, which enabled us to demonstrate that proviral load correlates not only with BLV infection capacity, as assessed by syncytium formation, but also with BLV disease progression. This study shows that, while CD5^+^ IgM^+^ B cells harbor the greatest BLV proviral load during the subclinical stage of EBL, CD4^+^ T cells and CD8^+^ T cells are also primary targets for BLV. Taken together, the results of this study show that the tropism of BLV is wider than previously thought.

## Abbreviations

BLV: Bovine leukemia virus; HTLV-1 and HTLV-2: Human T cell leukemia virus types 1 and 2; EBL: Enzootic bovine leucosis; AL: Aleukemic; PL: Persistent lymphocytosis; PBMC: Peripheral blood mononuclear cells; ELISA: Enzyme-linked immunosorbent assay; PCR: Polymerase chain reaction; CoCoMo: Coordination of common motifs; BoLA: Bovine leukocyte antigen; MAbs: Monoclonal antibodies; APC: Allophycocyanin; PE: Phycoerythrin.

## Competing interests

The authors declare no financial competing interests.

## Authors’ contributions

CJP and ST participated in performing real-time-PCR, flow cytometry, cell sorting and syncytium assay and in experimental design, analyzed data and drafted the manuscript. WCD contributed antibodies. TO and TN participated in performing the syncytium assay and sample collection. HI and MK participated in ELISA and sample collection. YA conceived the study, participated in experiments, participated in experimental design, coordinated experiments, and drafted the manuscript. All authors read and approved the final manuscript.

## Author information

CJ Panei (Ph.D.,D.V.M.): Visiting researcher of RIKEN and Assistant Professor of National University of La Plata. Shin-nosuke Takeshima (Ph.D.): ASI researcher of RIKEN and Associate Professor of The University of Tokyo. WC. Davis (Ph.D.,D.V.M.): Professor of Washington State University. Takashi Ohmori (Ph.D.,D.V.M.): Nippon Institute for Biological Science. Tetsuo Nunoya (Ph.D.,D.V.M.): Nippon Institute for Biological Science. Hiroshi Ishizaki (Ph.D.,D.V.M.): NARO Institute of Livestock and Grassland Sciences. Kazuhiro Matoba (M.A.): NARO Institute of Livestock and Grassland Sciences. Yoko Aida (Ph.D.,D.V.M.): Unit leader of RIKEN and Professor of The University of Tokyo.
